# The Influence of Fluid Intelligence, Executive Functions and Premorbid Intelligence on Memory in Frontal Patients

**DOI:** 10.3389/fpsyg.2018.00926

**Published:** 2018-06-08

**Authors:** Edgar Chan, Sarah E. MacPherson, Marco Bozzali, Tim Shallice, Lisa Cipolotti

**Affiliations:** ^1^Department of Neuropsychology, National Hospital for Neurology and Neurosurgery, London, United Kingdom; ^2^Institute of Neurology, University College London, London, United Kingdom; ^3^Centre for Cognitive Ageing and Cognitive Epidemiology, The University of Edinburgh, Edinburgh, United Kingdom; ^4^Human Cognitive Neuroscience, Department of Psychology, The University of Edinburgh, Edinburgh, United Kingdom; ^5^Neuroimaging Laboratory, Santa Lucia Foundation, Rome, Italy; ^6^Institute of Cognitive Neuroscience, University College London, London, United Kingdom; ^7^International School for Advanced Studies (SISSA-ISAS), Trieste, Italy

**Keywords:** frontal lobes, recall, recognition, memory intelligence, executive functions

## Abstract

**Objective:** It is commonly thought that memory deficits in frontal patients are a result of impairments in executive functions which impact upon storage and retrieval processes. Yet, few studies have specifically examined the relationship between memory performance and executive functions in frontal patients. Furthermore, the contribution of more general cognitive processes such as fluid intelligence and demographic factors such as age, education, and premorbid intelligence has not been considered.

**Method:** Our study examined the relationship between recall and recognition memory and performance on measures of fluid intelligence, executive functions and premorbid intelligence in 39 frontal patients and 46 healthy controls.

**Results:** Recall memory impairments in frontal patients were strongly correlated with fluid intelligence, executive functions and premorbid intelligence. These factors were all found to be independent predictors of recall performance, with fluid intelligence being the strongest predictor. In contrast, recognition memory impairments were not related to any of these factors. Furthermore, age and education were not significantly correlated with either recall or recognition memory measures.

**Conclusion:** Our findings show that recall memory in frontal patients was related to fluid intelligence, executive functions and premorbid intelligence. In contrast, recognition memory was not. These findings suggest that recall and recognition memory deficits following frontal injury arise from separable cognitive factors. Recognition memory tests may be more useful when assessing memory functions in frontal patients.

## Introduction

It is well-documented that frontal lobe lesions can result in memory difficulties (Wheeler et al., 1995; Kopelman, 2002). Memory impairments that result from frontal lobe lesions are thought to be distinct from pure amnesia, which arises from dysfunction of the diencephalon or temporal brain regions (Buckner et al., 1999). However, the exact nature of frontal lobe memory impairment is still somewhat unclear. For example, it is still debated whether frontal memory impairment manifests as a deficit in recall, recognition or both recall and recognition. Some argue that only recall memory is impaired while recognition memory remains relatively preserved (e.g., Janowsky et al., 1989; Milner et al., 1991). Others have reported impairments in both recall and recognition (e.g., Baldo et al., 2002; Alexander et al., 2003). Recently, memory performance in a large cohort of frontal patients was assessed using the Doors and People battery (Baddeley et al., 1994) which consists of verbal and visual recall and recognition tasks thought to be comparable in terms of difficulty (MacPherson et al., 2016). Frontal patients were found to be significantly impaired on both recall and recognition memory tasks compared to healthy controls. However, in line with the pattern of deficits found in an earlier meta-analysis (Wheeler et al., 1995), the effect sizes were greater for recall compared with recognition memory impairment, suggesting that recall memory is more affected following frontal lobe damage.

Although it is commonly thought that frontal memory impairments are secondary to impairment in executive processes, surprisingly few studies have directly examined the relationship between executive dysfunction and memory impairment. In list learning tasks, it is suggested that executive deficits in frontal patients cause a breakdown in top-down supervisory processes. This breakdown leads to the poor use of organizational strategies such as spontaneous categorization and semantic linkages during memory encoding, and poor search strategies and self-monitoring during memory retrieval (Baldo and Shimamura, 2002). An assumption then is that individuals with greater executive dysfunction will likely have greater memory deficits. Indeed, in the aging literature, it has been argued that memory difficulties in older adults are related to increased vulnerability to executive deficits due to age-related frontal–striatal changes (see Buckner, 2004 for a review). Executive functions have been shown to mediate the relationship between the effects of age and recall memory performance (Troyer et al., 1994; Crawford et al., 2000). Similarly, in early mild Alzheimer’s disease, recall memory performance has been shown to be correlated with performance on executive tasks (Baudic et al., 2006).

In patients with frontal lobe lesions, there has generally only been indirect support for the notion that memory impairments are related to executive deficits. A common finding is that word list-learning performance can be improved in frontal patients by explicitly grouping to-be-remembered words into semantic categories during encoding and by providing category cues during recall, thereby presumably reducing the ‘executive load’ of the task (e.g., della Rocchetta and Milner, 1993; Gershberg and Shimamura, 1995, but see Turner et al., 2007). Only a very few studies have explicitly examined the relationship between memory performance and performance on executive tasks in frontal patients. In one study, a correlation was found between recall memory performance (total number of words recalled) and phonemic fluency performance (FAS) in left dorsolateral frontal patients (Alexander et al., 2003). Interestingly, no similar correlation between fluency and recognition memory performance was found. However, no other executive measures were included in this study, limiting the conclusions that can be drawn.

Besides executive processes, more general cognitive processes may also contribute to memory performance in frontal patients. One prime candidate is fluid intelligence. Deficits in executive tasks in frontal patients have been argued to be underpinned by impairments in fluid intelligence (Duncan et al., 2000). In support of this, it has been shown that differences in performance on some executive tasks between frontal patients and healthy controls can be largely or entirely accounted for by performance on tests of fluid intelligence (Roca et al., 2010; Woolgar et al., 2010; Barbey et al., 2012; Keifer and Tranel, 2013). As such, it may be that memory difficulties in frontal patients might be better explained by impairment in fluid intelligence rather than executive functions. Indeed, fluid intelligence has been found to be the strongest predictor of episodic memory performance in healthy individuals (Aizpurua and Koutstaal, 2010).

We have also previously found that demographic factors such as age, years of education and premorbid intelligence, as measured by literacy attainment assessed using the National Adult Reading Test (NART IQ; Nelson and Willison, 1991), can significantly impact on executive impairments and fluid intelligence following frontal lobe injury (Cipolotti et al., 2015a; MacPherson et al., 2017). In a large cohort of frontal patients, we have shown that age and NART IQ are strongly correlated and predictive of performance on two executive tasks, verbal fluency and the Stroop Color Word test, over and above other factors such as lesion severity and chronicity. In addition, age, years of education and NART IQ are also related to fluid intelligence, though age seems to account for most of the unique variance. Indeed, age has been shown to exacerbate impairments in executive functions and fluid intelligence following frontal lesions (Cipolotti et al., 2015b). Whether these variables might also be related to, or mediate, memory performance following frontal lobe injury has yet to be investigated.

The aim of the current study was to increase our understanding of how executive processes relate to memory performance in patients with frontal lesions. Specifically, we wanted to examine the relationship between recall and recognition memory performance and age, education, premorbid intelligence, fluid intelligence and executive functions.

## Materials and Methods

### Participants

Thirty-nine patients (24 males, 15 females) with focal frontal lesions were prospectively recruited from the National Hospital for Neurology and Neurosurgery, Queen Square, London as part of two larger studies examining cognitive functions of the frontal lobe. Patients had an absence of psychiatric disorders, history of alcohol or substance abuse or previous neurological disorders. Frontal lesions were traced and classified by a neurologist who was blind to the study results based on MRI scans (or CT scans if MRI was unavailable). The aetiologies of the lesions were: glioma = 20; meningioma = 14; subarachnoid hemorrhage = 1; anterior communicating aneurysm = 3; and traumatic brain injury = 1. Importantly, we have previously shown that the grouping together of frontal patients with different aetiologies for the purposes of examining cognitive variables is methodologically justifiable (Cipolotti et al., 2015a). Sixteen patients had lesions confined to the left hemisphere, 18 patients to the right hemisphere and 5 patients had bilateral lesions. The majority of patients had lesions confined to the frontal lobes (*n* = 30; see Supplementary Table S1). The mean time since injury to assessment was 3.34 months (*SD* = 8.12 months). In addition, 46 healthy controls (HCs; 21 males, 25 females) with no history of neurological or psychiatric disorders were included for comparison. The study was approved by the National Hospital for Neurology and Neurosurgery and Institute of Neurology Joint Research Ethics Committee and written informed consent was gained according to the Declaration of Helsinki.

### Material and Procedure

#### Baseline Neuropsychological Assessment

All patients and HCs were assessed on a series of baseline neuropsychological measures. Premorbid level of optimal functioning (‘Premorbid intelligence’) was estimated using the National Adult Reading Test (NART; Nelson and Willison, 1991). Naming ability was assessed using the Graded Naming Test (GNT; McKenna and Warrington, 1983) and perceptual ability was assessed using the Incomplete Letters subtest from the Visual Object and Space Perception Battery (VOSP; Warrington and James, 1991).

#### Fluid Intelligence

Fluid intelligence was assessed using Raven’s Advanced Progressive Matrices (RAPM; Raven, 1976); an untimed, relatively culture-free, non-verbal test of abstract reasoning. The test requires the selection of the missing piece of a visual pattern from eight possible choices. The total number of correct responses in Set 1 (/12) was recorded and converted into age-adjusted scaled scores based on published norms.

#### Executive Functions – Verbal Fluency, Stroop Color Word Test

Two widely used neuropsychological tasks were administered to assess different aspects of executive functioning. These two tasks were chosen because they have been shown to require executive processes that are distinct from that which can be accounted for by fluid intelligence (Cipolotti et al., 2016; Cipolotti, unpublished). Verbal generation was assessed using the standard phonemic fluency test (‘FAS’; Benton and Hamsher, 1976). The total number of words recalled for all three letters, excluding errors (i.e., proper nouns or repetitions), was recorded. Verbal response inhibition was assessed using the Trenerry et al. (1989) version of the Stroop Color Word test which requires participants to name the ink color of 112 color words (e.g., say ‘Blue’ when the word Red is written in blue) printed on one A4 sheet. The time taken to read all 112 words was recorded in seconds.

#### Recall and Recognition Memory

All patients and HCs were assessed on a verbal list-learning recall memory test (‘Trieste Test’; Turner et al., 2007). Participants were asked to recall six 16-word lists that were each composed of four words from four different semantic categories (for further details on the construction of the word lists and semantic categories, see Turner et al., 2007). For each word list, words were either grouped according to their category (‘Blocked’) or they were mixed (‘Unblocked’). These two types of lists (Blocked or Unblocked) were presented in an alternating fashion across the task (i.e., blocked, unblocked, blocked etc…). For each 16-word list, each word was presented on a computer screen for 2 s with a 1 s interval between words. Following the list presentation, participants immediately completed a distractor task for 30 s (add 1 to a series of random numbers ranging from 1 to 99). Then, participants were asked to recall as many words as they could from the prior list (‘Uncued recall’). Once this was exhausted, the four semantic category labels were provided as prompts (e.g., jewels, occupations) for further recall (‘Cued recall’). The total number of words correctly recalled from each list before and after cueing was recorded, as well as separately for blocked and unblocked word lists. We also recorded the total number of errors made during recall (i.e., intrusions of words that were not presented).

A subset of frontal patients (*n* = 22) and HCs (*n* = 29) also completed the Doors and People Test battery (‘D&P’; Baddeley et al., 1994) which contained two recall tasks and two recognition tasks. Administration was conducted in accordance with procedures outlined in the manual. In brief, the verbal recall task required participants to learn and recall the names of four characters and their associated occupation, while in the visual recall task, participants had to copy and recall four simple line drawings. In both the verbal and visual recall tasks, participants were given three learning and recall trials. Points are awarded for recalled information across all three learning trials and the scores for the two recall tasks were combined to create an age-adjusted recall memory scaled score (‘D&P Recall’). For the recognition tasks, participants were asked to remember two sets of 12 stimuli presented for 3 s each; the targets were either male/female names in the verbal condition and photographs of different types of doors in the visual condition. Participants were then asked to recognize the target among three distractors. Points were awarded for each correctly identified target and combined to create an age-adjusted recognition memory scaled score (‘D&P Recognition’).

A second smaller subset of frontal patients (*n* = 15) also completed a 30-item three forced choice version (RMT-30) of the classic 50-item two forced choice Recognition Memory Test (Warrington, 1984). In the learning phase, participants were asked to remember 30 photographs of faces presented for 3 s each. Photographs were of unfamiliar Caucasian male faces with non-distinctive facial types. Participants were explicitly told to remember the faces and to decide whether the faces were ‘pleasant’ or ‘unpleasant’ to encourage encoding. In the recognition phase that immediately followed, target faces were presented again with two distractors each. The total number of targets correctly identified was recorded. Raw scores were converted to *z*-scores based on available normative data from a separate healthy control sample (see Supplementary Table S2).

### Statistical Analyses

Statistical analyses were carried out using IBM SPSS Statistics 22^1^. Firstly, we investigated differences between frontal patients and HCs, and between left and right frontal patients, on demographic variables and performance on baseline neuropsychological tests, measures of fluid intelligence and executive functions using independent samples *t*-tests for continuous variables and chi-square test for categorical variables. Performance differences on memory tasks between groups were examined using mixed-design repeated measures Analysis of Variance (ANOVA), except for RMT-30, where patient performance was evaluated using a one-sample *t*-test with a mean *z*-score of 0, as healthy control data were not available. An independent samples *t*-test was again used to compare differences between left and right frontal patients.

Secondly, we examined the relationship between recall and recognition memory performance and the different clinical and cognitive variables using two-tailed bivariate Pearson correlation analyses, for the frontal patients only.

Finally, for measures that were found to be significantly correlated with memory performance in our frontal patients, we ran a 3-stage hierarchical multiple regression to examine the independent predictive value of each variable. We chose a hierarchical approach because we were particularly interested in how executive functions predicted performance over and above any influences of general intelligence. Our previous work has shown that premorbid intelligence as measured by the NART is the best predictor of cognitive performance in frontal patients (e.g., MacPherson et al., 2017) and so this was entered in stage 1. Fluid intelligence was entered at stage 2 given that it has been argued to account for variance in executive deficits in frontal patients (e.g., Duncan et al., 2000). In stage 3, the two executive measures (Stroop Color Word test and verbal fluency) were entered together using a forced entry approach as we did not have an *a priori* hypothesis about the way in which each executive test might contribute to memory performance.

For results where *p*-values were less than 0.05, effect size and *r*-squared values were reported. For results where *p*-values were equal or greater than 0.05, additional Bayesian analyses were conducted where appropriate to determine the extent to which the odds were in favor of supporting the null-hypothesis (Gallistel, 2009). According to Jeffreys (1961), odds less than 3 are “weak,” odds between 3 and 10 are “substantial,” and odds between 10 and 100 are “strong.”

## Results

### Demographic and Baseline Neuropsychological Measures

Independent samples *t*-tests revealed that the frontal patient and HC groups did not significantly differ in terms of age (*p* > 0.1, *Odds* = 3.58), premorbid intelligence (*p* = 0.077, *Odds* = 1.81) and years of education (*p* > 0.1, *Odds* = 8.33; see **Table 1A**). Chi-squared analysis showed no significant difference in gender (*p* > 0.1). Patients were significantly poorer at naming than HCs [*t*(83) = 3.04, *p* < 0.01, *d* = 0.65] but there was no difference in performance on the test of visuo-perception (VOSP: *p* > 0.1, *Odds* = 8.23). Left and right frontal patients were well-matched on the demographic measures (*p* > 0.1; see **Table 1B**). There was also no difference in performance between left and right frontal patients on naming or visual perception (*p* > 0.1, *Odds* = 4.22 and *Odds* = 4.12, respectively).

**Table 1A:** Clinical and cognitive neuropsychological data for patients and healthy controls.

	Frontal			Healthy control		
	*n*	*M*	*SD*	*n*	*M*	*SD*
Age (years)	39	46.64	15.24	46	50.65	14.65
Education (years)	39	13.56	2.88	46	13.59	2.83
Premorbid Intelligence – NART (FSIQ)	37	107.65	13.04	44	112.09	9.19
Fluid Intelligence – Raven’s Advanced Progressive Matrices (Scaled Score)	37	10.97*	3.11	46	12.30	2.62
FAS (total words)	38	31.42**	15.41	46	49.80	12.81
Stroop Color-Word test (sec)	23	154.40**	53.22	33	125.23	27.76

*Difference between groups – ^∗^p < 0.05, ^∗∗^p < 0.01.*

**Table 1B:** Clinical and cognitive neuropsychological data for left and right hemisphere patients.

	Left frontal			Right frontal		
	*n*	*M*	*SD*	*n*	*M*	*SD*
Age (years)	16	47.19	13.09	18	46.06	15.73
Education (years)	16	14.19	2.48	18	13.56	3.09
Premorbid Intelligence – NART (FSIQ)	15	111.13	12.24	18	108.67	8.55
Fluid Intelligence – Raven’s Advanced Progressive Matrices (Scaled Score)	14	11.64	3.10	18	11.00	3.01
FAS (total words)	16	27.00*	15.68	17	38.18	13.80
Stroop Color-Word test (sec)	10	179.79**	52.45	10	114.28	19.87

*Difference between groups – ^∗^p < 0.05, ^∗∗^p < 0.01.*

### Fluid Intelligence and Executive Functions

Compared to HCs, the frontal patients had significantly lower scores on the test of fluid intelligence [*t*(81) = 2.11, *p* = 0.038, *d* = 0.46]. Not unexpectedly, the frontal group also performed significantly more poorly compared to HCs on the two measures of executive function – verbal fluency [*t*(82) = 5.97, *p* < 0.001, *d* = 1.30] and Stroop Color Word test [*t*(54) = 2.68, *p* = 0.01, *d* = 0.69]. **Table 1A** shows the mean scores for each of the tests for the two groups. The difference between patients and HCs remained significant when we co-varied for fluid intelligence (Verbal fluency: *p* < 0.001; Stroop Color Word test: *p* = 0.02).

Within the frontal group, no significant difference was found between left and right frontal patients on the test of fluid intelligence (*p* > 0.1, *Odds* = 4.01). In contrast, patients with left frontal lesions were found to generate significantly fewer words on verbal fluency [*t*(31) = -2.18, *p* = 0.037, *d* = 0.76] and were slower on the Stroop Color Word test compared with patients with right frontal lesions [*t*(18) = 3.69, *p* = 0.002, *d* = 1.65]. The difference between left and right frontal patients remained significant when we co-varied for fluid intelligence (Verbal fluency: *p* = 0.021; Stroop Color Word test: *p* = 0.002). **Table 1B** shows the mean scores for each of the tests for the two groups.

### Recall Memory

Performance on the Trieste test of verbal list-learning was examined using a mixed-design repeated measures Analysis of Variance (ANOVA) with 2 within-subjects factors of Block (Blocked, Unblocked) and Cue (Cue, Uncued) and 1 between-subjects factor of Group (Patients, HCs). There was a significant main effect of Group in which patients recalled fewer words than HCs [*F*(1,83) = 6.90, *p* = 0.01, $\eta_{p}^{2}$ = 0.08]. There was a significant main effect of Block [*F*(1,83) = 10.63, *p* = 0.002, $\eta_{p}^{2}$ = 0.67] and Cue [*F*(1,83) = 170.52, *p* < 0.001, $\eta_{p}^{2}$ = 0.11] showing that word-lists that were semantically blocked during presentation and providing cues improved recall performance. Crucially, however, there was no significant interaction between either factors with Group (Patients or HCs; *p* > 0.1). That is, frontal patients did not significantly benefit from blocking or cueing more than HCs (see **Table 2A**). There was no significant difference in the number of recall errors made between the frontal patients [*M (SD)* = 5.69 (3.95)] and HCs [(*M (SD)* = 4.70 (4.25)].

**Table 2A:** Recall and Recognition memory performance for patients and healthy controls.

	Frontal			Healthy control		
	*n*	*M*	*SD*	*n*	*M*	*SD*
Trieste Test	*n* = 39			*n* = 46		
Total Correct (/96)		60.67**	19.00		69.76	13.58
Blocked + Uncued (/48)		26.62	10.80		31.59	8.67
Blocked + Cued (/48)		31.77	9.65		35.87	6.57
Unblocked + Uncued (/48)		24.23	11.84		30.15	9.75
Unblocked + Cued (/48)		28.95	9.93		33.85	8.05
Doors and People test (D&P)	*n* = 22			*n* = 29		
D&P Recall (SS)		9.00**	2.86		11.48	3.40
Verbal (SS)		8.35	4.04		11.03	3.91
Visual (SS)		9.87	2.24		11.38	2.87
D&P Recognition (SS)		10.09*	3.13		11.93	2.83
Verbal (SS)		10.78	3.49		12.66	3.06
Visual (SS)		8.57	3.09		10.38	3.05
RMT-30	*n* = 15					
RMT-30 (*z*-score)		-0.67*	1.11	–	–	–

*Difference between groups – ^∗^p < 0.05, ^∗∗^p < 0.01.*

Recall performance on the Doors and People test was examined using a mixed-design repeated measures ANOVA with 1 within subjects factor of domain (verbal, visual) and 1 between-subjects factor of Group (Patients, HC). Frontal patients scored significantly more poorly compared to healthy controls overall [*F*(1,50): 7.71, *p* = 0.008, $\eta_{p}^{2}$ = 0.13]. There was no significant effect of domain (*p* = 0.1) and no interaction between domain and group (*p* > 0.1), suggesting that performance on the two recall subtests were relatively comparable.

Within the frontal group, there was no significant difference in the total words recalled on the Trieste test between patients with left and right sided lesions (*p* > 0.1, *Odds* = 1.69) and on D&P Recall (*p* > 0.1, *Odds* = 3.14; see **Table 2B**).

**Table 2B:** Recall and recognition memory performance for left and right hemisphere patients.

	Left frontal			Right frontal		
	*n*	*M*	*SD*	*n*	*M*	*SD*
Trieste Test	*n* = 16			*n* = 18		
Total Correct/96		59.75	18.42		65.61	16.18
Doors and People test (D&P)	*n* = 9			*n* = 11		
D&P Recall (SS)		9.33	2.30		9.18	3.22
D&P Recognition (SS)		10.11	3.89		10.54	2.62
RMT-30	*n* = 6			*n* = 7		
RMT-30 (*z*-score)		-0.82	0.10		-0.31	1.23

### Recognition Memory

Recognition performance on the Doors and People test was examined using a mixed-design repeated measures ANOVA with 1 within subjects factor of domain (verbal, visual) and 1 between-subjects factor of Group (Patients, HC). Frontal patients scored significantly more poorly compared to healthy controls overall [*F*(1,50) = 6.85, *p* = 0.012, $\eta_{p}^{2}$ = 0.12]. There was a significant effect of domain [*F*(1,50) = 17.75, *p* < 0.01, $\eta_{p}^{2}$ = 0.26] which showed that the visual recognition test was significantly harder overall [*M (SD)* = 9.47 (0.43)] compared with the verbal recognition memory test [*M (SD)* = 11.71 (0.46)]. However, there was no significant interaction between domain and group (*p* > 0.1).

On the RMT-30, *z*-score performance of frontal patients was assessed using a one-sample *t*-test (Mean *z*-score = 0). Mean *z*-score performance of the frontal patients was statistically different from zero [*t*(14) = -2.32, *p* = 0.036, *d* = 0.60].

Within the frontal group, as with recall performance, there was no significant difference on D&P Recognition between patients with left and right sided lesions (*p* > 0.1, *Odds* = 3.20) and on RMT-30 (*p* > 0.1, *Odds* = 1.30).

### Relationship Between Memory Performance and the Clinical and Cognitive Variables

We conducted two-tailed bivariate Pearson correlation analyses to examine the relationship between recall and recognition memory performance in frontal patients and their clinical and cognitive variables. Given the lack of significant difference in performance between the left and right frontal patients on all memory measures, the two groups were combined in all correlation and regression analyses to increase power. To reduce the likelihood of false-positives, only the main memory measures that were found to be meaningfully impaired compared with healthy controls were included in the analysis; the Trieste test Total Score, D&P Recall, D&P Recognition, and the RMT-30. Clinical variables included were age, years of education and premorbid intelligence as assessed by the NART. Cognitive variables included were fluid intelligence as measured by Raven’s Progressive Matrices and the two executive measures of verbal fluency and the Stroop Color Word test.

**FIGURE 1:**
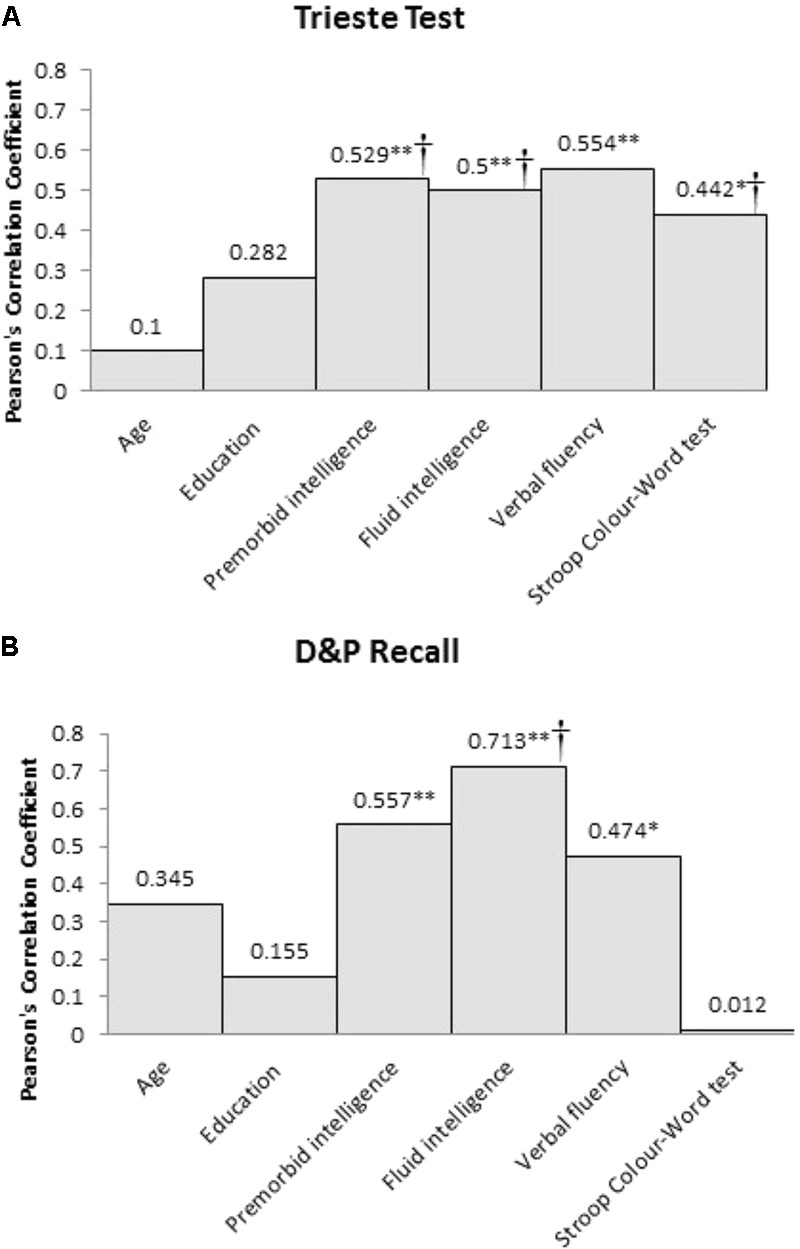
Pearson’s correlation coefficient (*r*) between the two recall memory measures, **(A)** Trieste Test and **(B)** D&P Recall, and the clinical and cognitive variables for frontal patients (^∗^Sig. correlation, *p* < 0.05, ^∗∗^Sig. correlation, *p* < 0.01). ^†^Significant predictor of memory performance in the hierarchical multiple regression.

Both recall memory measures were significantly correlated with premorbid intelligence (Trieste test, *p* = 0.001; D&P Recall, *p* = 0.007), fluid intelligence (Trieste test, *p* = 0.002; D&P Recall, *p* < 0.001), and verbal fluency (Trieste test, *p* < 0.001; D&P Recall, *p* = 0.026). Only performance on the Trieste test was related to verbal response inhibition as assessed using the Stroop test (*p* = 0.035) but not D&P Recall (*p* > 0.1). Performance on the two recall measures were significantly correlated with each other (*p* < 0.001). Neither recall memory measures were correlated with age or years of education. The absolute Pearson’s correlation coefficient between the two recall memory measures and the clinical and cognitive variables are shown in **Figure 1**.

**FIGURE 2:**
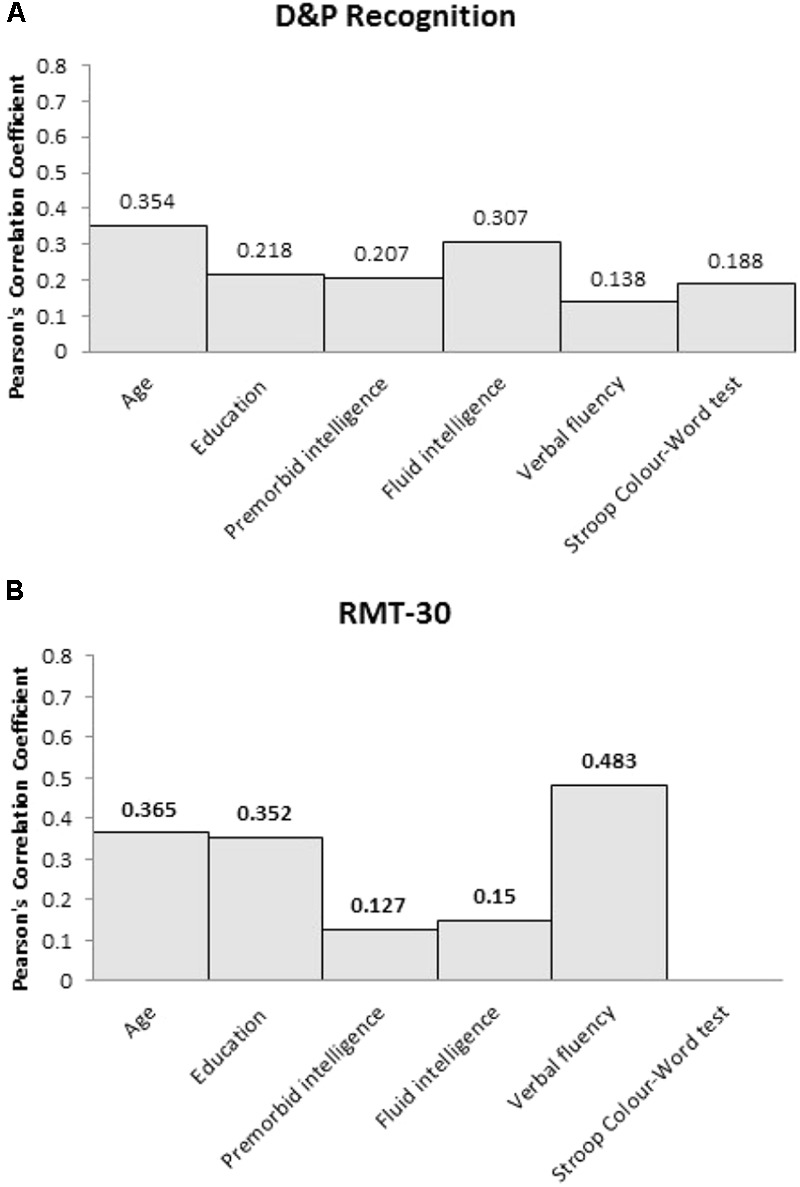
Pearson’s correlation coefficient (*r*) between the two recognition memory measures, **(A)** D&P Recognition and **(B)** RMT-30, and the clinical and cognitive variables for frontal patients.

In contrast, neither recognition memory measures, D&P Recognition or RMT-30, were correlated with premorbid intelligence (*p* > 0.1), fluid intelligence (*p* > 0.1) or either executive measures (see **Figure 2**). Neither recognition memory measures were correlated with age or years of education.

### Predictors of Recall Memory Performance

Given that the recall memory measures were significantly correlated with premorbid intelligence, fluid intelligence, and performance on the executive tasks, we examined the relative predictive value of these three variables using a 3-stage hierarchical multiple regression. Premorbid intelligence was entered at stage 1, fluid intelligence was entered at stage 2 and the two executive tasks (Stroop Color Word test and verbal fluency) were entered at stage 3.

Using performance on Trieste Total Recall as the dependent variable, the hierarchical multiple regression revealed that at stage 1, premorbid intelligence contributed significantly to the regression model [*F*(1,22) = 11.76, *p* < 0.01] and accounted for 36% of the variance in recall memory performance. Introducing fluid intelligence at stage 2 explained an additional 26% of the variation, explaining a total of 62% variance in recall memory performance, and this change in *R*^2^ was significant [*F*(1,20) = 13.57, *p* < 0.01]. Adding the two executive tasks explained an additional 19% of the variance to the model and this change was significant [*F*(2,18) = 8.82, *p* < 0.01]. The final model accounted for 81% of the variance in Trieste Total Recall [*F*(4,12) = 18.83, *p* < 0.01]. Premorbid intelligence (β = -0.45, *p* = 0.041), fluid intelligence (β = 1.24, *p* < 0.01), and Stroop Color Word test (β = -0.59, *p* < 0.01) were all significant predictors whereas verbal fluency was not (*p* > 0.1).

The same analysis was repeated with D&P Recall as the dependent variable. The hierarchical multiple regression revealed that at stage 1, premorbid intelligence contributed significantly to the regression model [*F*(1,19) = 7.73, *p* = 0.012] and accounted for 29% of the variance in recall memory performance. Introducing fluid intelligence at stage 2 explained an additional 20% of the variation, explaining a total of 49% variance in recall memory performance, and this change in *R*^2^ was significant [*F*(1,18) = 7.09, *p* = 0.016]. Unlike Trieste Total Recall, adding the two executive tasks did not significantly add to the variance explained by the model for D&P Recall performance (*p* > 0.1). At stage 2, only fluid intelligence (β = 0.85, *p* = 0.016) was a significant predictor of recall performance, whereas premorbid intelligence was not (*p* > 0.1).

As different variables were found to be significant predictors of recall memory performance in frontal patients, we examined whether the difference in performance originally found between frontal patients and healthy controls could be accounted for by these predictors by entering them as covariates using an Analyses of Covariance. The difference in performance between frontal patients and healthy controls on Trieste Total Recall was no longer significant once premorbid intelligence, fluid intelligence and Stroop Color Word test were entered as covariate (*F*(1,51) = 0.053, *p* = 0.819). In contrast, the difference in performance between frontal patients and healthy controls on D&P Recall remained significant when fluid intelligence was entered as a covariate (*F*(1,49) = 6.38, *p* = 0.015).

Given that recognition memory performance was not correlated with any of the clinical or cognitive variables, multiple regression was not performed.

## Discussion

For the first time, we investigated how demographic factors of age and education, premorbid intelligence, fluid intelligence, and executive functions relate to and account for recall and recognition memory performance in frontal patients. Our frontal patients were found to be impaired on two different measures of recall memory and two different measures of recognition memory compared with healthy controls. This finding supports previous suggestions that frontal injury can result in both recall and recognition memory deficits (e.g., MacPherson et al., 2016). Crucially, however, we show that the nature of these deficits may be separable in how they relate to other clinical and cognitive factors.

For recall memory, performance in frontal patients on both recall memory measures was correlated with premorbid intelligence, fluid intelligence, and verbal fluency. Performance on the list learning task was also related to the Stroop Color Word test. Investigation into the individual contributions of premorbid intelligence, fluid intelligence and executive functions on predicting recall memory performance revealed slightly different but converging results for our two measures. For the Trieste list-learning task, all three variables were significant independent predictors of recall performance. Of the executive tasks, although both verbal fluency and Stroop Color Word were correlated with performance, only the Stroop Color Word test was a significant predictor of performance when all variables were taken into account. Of all the significant predictors, fluid intelligence was the strongest predictor of performance. For D&P Recall, fluid intelligence was the only significant predictor of recall performance. Despite D&P Recall performance being correlated with both premorbid intelligence and verbal fluency, neither variable contributed significantly over and above the variance accounted for by fluid intelligence. Overall, our findings suggest that recall memory deficits in frontal patients are best accounted for by fluid intelligence. The difference in findings between our two recall measures might reflect inherent differences in the two measures. The Trieste list learning task has 16-items per word list and one learning trial per list whereas the D&P Recall tasks only contain 4-items and have three repeated learning trials. Thus, it may be that the Trieste test requires greater demand on supervisory processes such as strategy and inhibition to encode the multiple word lists efficiently and avoid interference across lists (Baldo and Shimamura, 2002). However, investigation into the differences between the demands of the tasks warrants further study.

The finding that recall memory in frontal patients is related to fluid intelligence processes is in keeping with a specific theoretical proposal regarding the neurocognitive architecture of the frontal lobe. Fluid intelligence is taken as a measure of some general or *g* factor that can broadly account for performance across a range of different tasks (Duncan et al., 2000). It captures the mental processes required for breaking tasks down into subcomponents that are thought to be necessary to perform most cognitive tasks, particularly novel or complex ones. It has been argued that fluid intelligence can be mapped onto a multiple-demand (MD) network in the brain that involves predominantly frontal–parietal regions (Woolgar et al., 2010). As such, damage to frontal brain regions often results in impairment in fluid intelligence (Duncan et al., 2000). It has been shown that fluid intelligence can account for some executive deficits that result from frontal lobe injury (Roca et al., 2010). Furthering this, our data suggests that impairment in fluid intelligence following frontal lesions may also account for performance in recall memory tasks.

Recall performance in frontal patients was also correlated with premorbid intelligence as assessed by the NART but not years of education. NART was also a significant independent predictor of Trieste performance. Both NART and years of education are often thought of as comparable indicators of premorbid intelligence. However, we have recently shown that these two variables do not represent the same proxy measure, at least following frontal injury, with NART being a better predictor of executive functions (MacPherson et al., 2017). Our findings further extend the important role of premorbid intelligence as measured by the NART in protecting against the impact of frontal brain injury on memory functions.

Recall memory impairment in our frontal patients was correlated with impairment in executive processes. Consistent with Alexander et al. (2003), we found that recall, but not recognition memory was related to performance on verbal fluency. In addition, Trieste recall was also related to response inhibition as measured by the Stroop Color Word Test. As far as we know, this is the first time in which the contribution of different executive measures to recall memory in frontal patients has been examined independently. Previous work has generally combined different executive measures into a composite, thereby limiting the potential for differences between tests to be explored (e.g., Troyer et al., 1994; Crawford et al., 2000). In our study, although both verbal fluency and Stroop performance were correlated with recall, only performance on the Stroop, but not verbal fluency, was a significant predictor independent of premorbid intelligence and fluid intelligence. Our findings show that different executive functions may contribute to recall performance differently. Furthermore, our findings support the notion that some executive abilities are dissociable from fluid intelligence following frontal injury (Cipolotti et al., 2016; Cipolotti, unpublished). In future, it would be important to consider this in further detail with a wider variety of tasks tapping different known executive functions.

In contrast to recall memory, performance on recognition memory measures in our frontal patients were not significantly related to premorbid intelligence, fluid intelligence or either executive measure. Importantly, however, frontal patients were significantly impaired on the recognition memory measures, which is consistent with previous findings (Wheeler et al., 1995; MacPherson et al., 2016). The lack of relationship between recognition memory impairment and performance on other cognitive tests suggests that recognition memory performance is dissociated from premorbid intelligence, fluid intelligence and executive functions. It may be that poor performance on the recognition memory task reflects some genuine deficit in memory processes (Cipolotti et al., 2001). Alternatively, it has been shown that poor recognition performance in frontal patients may be related to specific impairment in familiarity judgments; a difficulty in frontal patients to inhibit responding ‘yes’ to similar distractors (Alexander et al., 2003; MacPherson et al., 2008).

We did not find any significant relationship between performance on any of our memory measures and patients’ age. In our previous work, we have demonstrated that age predicts performance on executive tasks in frontal patients (MacPherson et al., 2017) and modulates the magnitude of their impairment, whereby middle-aged and older frontal patients had exacerbated executive impairment compared to younger adults (Cipolotti et al., 2015b). This latter effect was not found for performance on non-executive tasks that do not rely on frontal functions. The lack of relationship between age and memory performance in our current study appears inconsistent also with what is shown in the healthy and pathological aging literature (Buckner, 2004). It may be that the impact of frontal lesions decompensates for any premorbid relationship between age and memory performance (but see Cipolotti et al., 2015b).

Our study represents a first step into exploring the relationship between memory performance and fluid intelligence, executive functions and premorbid intelligence in frontal patients. Given our findings, it would be important to examine these underlying mechanisms further in a larger sample of frontal patients to allow for grouping of patients into different subregions and more detailed examination of neuropathological factors such as proportionate gray matter loss or white matter tract involvement. It has been shown that the pattern of memory impairment may vary depending on the frontal subregion injured consistent with the known specialization of function in different frontal areas (Stuss and Alexander, 2005; Turner et al., 2007). It may be that factors such as premorbid intelligence and fluid intelligence impact upon recall performance across frontal subregions whereas different executive functions have a more location-specific effect. Furthermore, our slightly different pattern of findings across our two recall memory tasks suggests a more systematic exploration of frontal memory processes is necessary to further examine the different influences of fluid intelligence and executive tasks on recall task demands.

Overall, we have shown that recall memory performance in frontal patients can largely be accounted for by fluid intelligence, executive functions and premorbid intelligence. Future studies examining memory performance in frontal patients should consider how these factors might mediate any deficits observed. Although all three variables were related to recall memory performance, general fluid intelligence appears to be the strongest predictor. This was not replicated in recognition memory performance. Our findings suggest that it may be more meaningful to assess memory functions in frontal patients using recognition memory, as recall performance may likely be affected by non-memory related processes.

## Author Contributions

All authors were involved in the conception of the study. EC and SM were involved in the collection of the data. EC, SM, and MB were involved in the analyses of the data. EC, SM, and LC were involved in the writing and editing of the manuscript. MB and TS reviewed the manuscript.

## Conflict of Interest Statement

The authors declare that the research was conducted in the absence of any commercial or financial relationships that could be construed as a potential conflict of interest.

## Footnotes

1 https://www.ibm.com/products/spss-statistics
