# Supplementary material for: The Influence of Fluid Intelligence, Executive Functions and Premorbid Intelligence on Memory in Frontal Patients
Source: Front Psychol. 2018 Jun 8;9:926. doi: 10.3389/fpsyg.2018.00926 (PMC6002504; doi:10.3389/fpsyg.2018.00926)
Supplement: Supplementary file 1 [file Table_1.DOCX]

|  |  | LEFT FRONTAL | | | RIGHT FRONTAL | | | | SUBCORTICAL | NON-FRONTAL |  |
| --- | --- | --- | --- | --- | --- | --- | --- | --- | --- | --- | --- |
|  |  | Orbital | Medial | Lateral | | Orbital | Medial | Lateral |  |  |  |
| **Left Group** | |  |  |  | |  |  |  |  |  |  |
| AC | Glioma |  | **●** | **●** | |  |  |  |  |  |  |
| ED | Glioma | **●** | **●** |  | |  |  |  |  |  |  |
| GS | Glioma |  |  | **●** | |  |  |  |  |  |  |
| GT | Glioma |  | **●** |  | |  |  |  |  |  |  |
| GY | Glioma |  | **●** | **●** | |  |  |  |  |  |  |
| PF | Glioma |  | **●** |  | |  |  |  |  |  |  |
| PS | Glioma |  |  | **●** | |  |  |  |  |  |  |
| DW | Glioma | **●** | **●** | **●** | |  |  |  | **●** |  |  |
| AA | Glioma |  | **●** | **●** | |  |  |  | **●** | **●** |  |
| LA | Meningioma |  | **●** | **●** | |  |  |  |  |  |  |
| MJ | Meningioma |  | **●** | **●** | |  |  |  |  |  |  |
| NH | Meningioma |  |  | **●** | |  |  |  |  |  |  |
| JF | Meningioma |  | **●** | **●** | |  |  |  | **●** |  |  |
| DS | Meningioma |  |  | **●** | |  |  |  |  | **●** |  |
| VB | Meningioma | **●** | **●** |  | |  |  |  |  |  |  |
| EH | SAH |  |  | **●** | |  |  |  |  |  |  |
| **Right Group** | |  |  |  | |  |  |  |  |  |  |
| DL | Glioma |  |  |  | |  | **●** |  |  |  |  |
| JS | Glioma |  |  |  | |  | **●** | **●** |  |  |  |
| JW | Glioma |  |  |  | |  | **●** |  |  |  |  |
| KR | Glioma |  |  |  | |  | **●** |  |  |  |  |
| SK | Glioma |  |  |  | | **●** | **●** | **●** |  |  |  |
| SL | Glioma |  |  |  | |  | **●** |  |  |  |  |
| SV | Glioma |  |  |  | |  | **●** |  |  |  |  |
| BB | Glioma |  |  |  | | **●** | **●** | **●** | **●** |  |  |
| EH | Glioma |  |  |  | |  |  | **●** |  |  |  |
| PI | Glioma |  |  |  | |  |  | **●** |  |  |  |
| SC | Glioma |  |  |  | |  | **●** | **●** | **●** |  |  |
| CF | Meningioma |  |  |  | | **●** | **●** |  |  |  |  |
| TR | Meningioma |  |  |  | | **●** |  |  |  |  |  |
| AB | Meningioma |  | **●** |  | | **●** |  | **●** | **●** |  |  |
| FM | Meningioma |  |  | **●** | |  | **●** |  |  |  |  |
| MK | Meningioma |  |  |  | |  | **●** | **●** |  |  |  |
| RD | ACoAA |  |  |  | | **●** |  |  |  |  |  |
| SH ACoAA | |  |  |  | |  | **●** |  |  |  |  |
| **Bilateral Group** | |  |  |  | |  |  |  |  |  |  |
| RP | Meningioma | **●** | **●** |  | | **●** |  |  |  |  |  |
| PF | Meningioma | **●** | **●** |  | | **●** | **●** |  | **●** |  |  |
| AB | Meningioma | **●** | **●** | **●** | | **●** | **●** | **●** | **●** | **●** |  |
| MW | ACoAA | **●** |  |  | | **●** |  |  |  |  |  |
| MC | TBI | **●** | **●** |  | | **●** | **●** | **●** |  |  |  |

**Supplementary Table 1.** Lesion Location and Aetiology of Frontal Patients**.**

Dots indicate the brain region that is involved in the lesion. SAH: Subarachnoid Haemorrhage; ACoAA: Anterior Communicating Artery Aneurysm; TBI: Traumatic Brain Injury.
